# Team Strategies and Tools to Enhance Performance and Patient Safety (TeamSTEPPS) to Improve Collaboration in School Mental Health: Protocol for a Mixed Methods Hybrid Effectiveness-Implementation Study

**DOI:** 10.2196/26567

**Published:** 2021-02-08

**Authors:** Aparajita Kuriyan, Grace Kinkler, Zuleyha Cidav, Christina Kang-Yi, Ricardo Eiraldi, Eduardo Salas, Courtney Benjamin Wolk

**Affiliations:** 1 Department of Psychiatry Perelman School of Medicine University of Pennsylvania Philadelphia, PA United States; 2 Leonard Davis Institute for Health Economics University of Pennsylvania Philadelphia, PA United States; 3 Department of Pediatrics Perelman School of Medicine University of Pennsylvania Philadelphia, PA United States; 4 Department of Child and Adolescent Psychiatry and Behavioral Sciences Children’s Hospital of Philadelphia Philadelphia, PA United States; 5 Department of Psychological Sciences Rice University Houston, TX United States

**Keywords:** teams, Team Strategies and Tools to Enhance Performance and Patient Safety, school mental health, school health

## Abstract

**Background:**

Public schools in the United States are the main providers of mental health services to children but are often ill equipped to provide quality mental health care, especially in low-income urban communities. Schools often rely on partnerships with community organizations to provide mental health services to students. However, collaboration and communication challenges often hinder implementation of evidence-based mental health strategies. Interventions informed by team science, such as Team Strategies and Tools to Enhance Performance and Patient Safety (TeamSTEPPS), have the potential to improve treatment implementation and collaboration within schools.

**Objective:**

The objective of this study is to improve communication and collaboration strategies among mental health and school staff by adapting an evidence-based team science intervention for school settings. We present a protocol for a hybrid effectiveness-implementation study to adapt TeamSTEPPS using stakeholder feedback, develop a tailored implementation plan, and pilot the adapted content in eight schools.

**Methods:**

Study participants will be recruited from public and charter schools and agencies overseeing school mental health services in the local metro area. We will characterize current services by conducting a needs assessment including stakeholder interviews, observations, and review of administrative data. Thereafter, we will establish an advisory board to understand challenges and develop possible solutions to guide additional TeamSTEPPS adaptations along with a complementary implementation plan. In aim 3, we will implement the adapted TeamSTEPPS plus tailored implementation strategies in eight schools using a pre-post design. The primary outcome measures include the feasibility and acceptability of the adapted TeamSTEPPS. In addition, self-report measures of interprofessional collaboration and teamwork will be collected from 80 participating mental health and school personnel. School observations will be conducted prior to and at three time points following the intervention along with stakeholder interviews. The analysis plan includes qualitative, quantitative, and mixed methods analysis of feasibility and acceptability, school observations, stakeholder interviews, and administrative data of behavioral health and school outcomes for students receiving mental health services.

**Results:**

Recruitment for the study has begun. Goals for aim 1 are expected to be completed in Spring 2021.

**Conclusions:**

This study utilizes team science to improve interprofessional collaboration among school and mental health staff and contributes broadly to the team science literature by developing and specifying implementation strategies to promote sustainability. Results from this study will provide knowledge about whether interventions to improve school culture and climate can ready both mental health and school systems for implementation of evidence-based mental health practices.

**Trial Registration:**

ClinicalTrials.gov NCT04440228; https://clinicaltrials.gov/ct2/show/NCT04440228

**International Registered Report Identifier (IRRID):**

DERR1-10.2196/26567

## Introduction

### Background

Youth living in poverty experience internalizing and externalizing mental health disorders at considerably higher rates than higher socioeconomic status peers. One in five children living below 100% of the federal poverty level has a mental, behavioral, or developmental disorder [1]. These disproportionately high rates are due in large part to exposure to psychosocial stressors such as community violence and housing insecurity [2,3]. Approximately half of all children with emotional and behavioral disorders receive mental health services [4]; however, rates of service utilization are likely lower among low-income youth [5,6]. Public schools have become the main provider of mental health services to children in the United States and offer a way to increase access for low-income youth [7]. In fact, a 2017-2018 national survey showed that 58% of urban schools provided mental health diagnostic services and 42% provided treatment [8]. However, the primary mission of schools is education, not health care, and many schools are ill equipped to provide quality mental health care to students [9,10].

Due to a lack of internal capacity to adequately meet student mental health needs, districts often have contracts with community agencies for services. A comprehensive national survey found that about half of school districts in the United States had contracts with community organizations for student mental health services, typically provided in school with a combination of school and community staff [11]. Regarding the coordination of mental health activities in schools, about one-third of schools never held interdisciplinary staff meetings, and the most frequent form of coordination was informal communication [11]. Although school-based mental health services fill an unmet need in an accessible context for children, it is unclear whether services are coordinated effectively for maximal positive impact on student outcomes while minimizing burden on schools.

Effective collaboration among interdisciplinary personnel is a necessary yet understudied aspect of providing school-based mental health services. Supporting school-aged children with mental health needs often requires a team of providers, including teaching and nonteaching staff, paraprofessionals, mental health clinicians, case managers, and physicians [12]. Collaboration challenges include limited time and resources, poor communication, and vaguely defined roles [13-18]. For example, mental health teams and teachers may not realize the mutual benefit of receiving each other’s input and collaboration on discipline-specific yet shared goals for classroom behavior management or treatment plans. Even when teachers are interested in increasing collaboration and involvement in student mental health [19], specific strategies for facilitating this engagement are lacking. Ineffective teamwork may hinder the quality of services as evidence-based school mental health interventions typically rely on the engagement and coordination of multiple individuals [20].

Team science literature has the potential to inform implementation efforts and improve collaboration within school-based mental health teams [21-23]. One particular team training intervention, Team Strategies and Tools to Enhance Performance and Patient Safety (TeamSTEPPS) [24,25], has been widely used in health care settings with encouraging outcomes [26,27]. TeamSTEPPS has been associated with improvements in teamwork and communication [26,28], reduced provider burnout [29] and turnover [30], and improved patient outcomes [31]. Core competencies targeted in TeamSTEPPS are leadership, situation monitoring, mutual support, and communication. These competencies represent trainable skills [23], and performance, knowledge, and attitudinal outcomes result from proficiency in these competencies. The curriculum consists of an introductory module and four didactic modules targeting each core competency [23-25]. Defining team skills, demonstrating strategies for improving proficiency in competencies, and identifying tools for overcoming barriers are emphasized [23]. Vignettes and case scenarios reinforce learning. TeamSTEPPS implementation typically occurs as a multiphase process that includes (1) assessment, (2) planning, training, and implementation, and (3) sustainment, though the necessary implementation supports have not been well defined in the literature. Improvements in team skills and behaviors of staff, such as those that have been attributed to TeamSTEPPS [26,28], have the potential to improve culture and climate in schools, which may lead to improved student outcomes [32].

### Prior Work

As TeamSTEPPS was developed for health care settings, it requires modifications for the school mental health context. Our team has previously adapted the intervention for mental health teams working in schools and has examined the feasibility and acceptability of the adaptation [33]. Relevant stakeholders from school mental health teams employed by community organizations advised the adaptation, and school staff did not participate. Core TeamSTEPPS content remained largely unchanged in the adaptation because participants reported it was relevant as is; however, we adapted language throughout to be consistent with preferred nomenclature in schools (eg, “patients” was changed to “students” or “children”) and revised case examples and vignettes with ones informed by community partners’ experiences. Teams in six schools were randomized to receive the adapted TeamSTEPPS approach or usual supports. The results indicated that TeamSTEPPS was feasible and acceptable for implementation, and leadership emerged as an important facilitator. Barriers to implementation success included staff turnover, lack of resources, and challenges in the school-mental health team relationship [34]. Overall, this preliminary work suggested that TeamSTEPPS was promising for school mental health teams. Stakeholder feedback indicated that more robust supports, such as ongoing consultation and booster training, would enhance implementation efforts. Additionally, engaging school personnel directly in further adapting TeamSTEPPS was highlighted as an important next step. This study builds upon our previous findings by further adapting TeamSTEPPS in collaboration with school personnel, and defining and piloting implementation supports.

### Current Study

**Figure 1:**
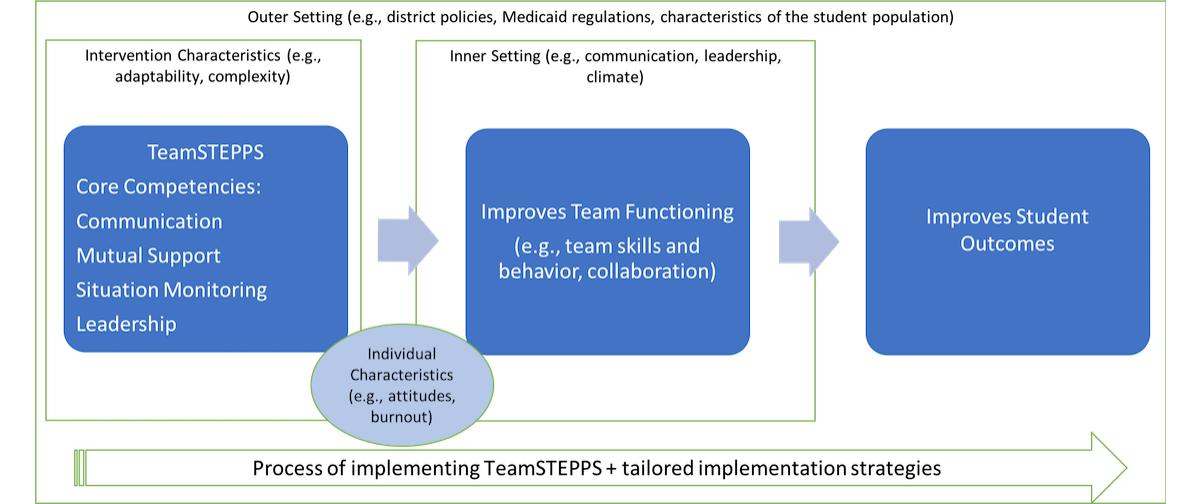
Guiding conceptual model. TeamSTEPPS: Team Strategies and Tools to Enhance Performance and Patient Safety.

This study will expand the adaptation of TeamSTEPPS using additional stakeholder feedback, develop a tailored implementation plan, and pilot the new adapted version. The Consolidated Framework for Implementation Research (CFIR) provides an overarching framework for the project to guide implementation (Figure 1). The CFIR, synthesized from the health services implementation literature, represents “an overarching typology - a list of constructs to promote theory development and verification about what works where and why across multiple contexts” [35]. The five major domains of the CFIR include intervention characteristics, outer setting, inner setting, characteristics of individuals involved, and implementation process. Complementary to the CFIR, the School Implementation Strategies, Translating Expert Recommendations for Implementing Change (ERIC) Resources (SISTER) [36] will be a guide for tailoring implementation strategies. The SISTER is a compilation of implementation strategies adapted from the ERIC taxonomy, specifically for schools.

The specific aims of this project are to (1) characterize the use of school mental health services in urban districts within the United States, including challenges and successes, using multiple sources of information; (2) identify interorganizational challenges and required components of TeamSTEPPS to adapt (the product of aim 2 will be an adapted TeamSTEPPS for both school mental health and school-employed personnel, and specific tailored implementation strategies to improve services in schools in conjunction with TeamSTEPPS [TeamSTEPPS plus tailored implementation plan]); and (3) explore the feasibility, acceptability, and impact of TeamSTEPPS plus tailored implementation strategies on interprofessional collaboration, teamwork, and student outcomes. Primary implementation outcomes [37] of interest are the feasibility and acceptability of the adapted TeamSTEPPS. Secondary goals include exploring the impact of TeamSTEPPS plus tailored implementation strategies on interprofessional collaboration, teamwork, and student outcomes.

This study has a mixed methods effectiveness-implementation hybrid design. The effectiveness of TeamSTEPPS will be examined while knowledge of implementation barriers and facilitators are incorporated into an implementation plan [38].

## Methods

### Overview

**Figure 2:**
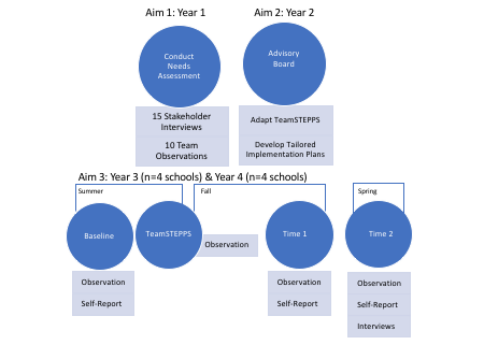
Overall study timeline. TeamSTEPPS: Team Strategies and Tools to Enhance Performance and Patient Safety.

Figure 2 presents an overall timeline for the study. The objective in year 1 is to better understand the needs of our district partners. The objective in year 2 is to adapt TeamSTEPPS and co-develop a tailored implementation plan with school and mental health team partners. During years 3 and 4, we will pilot test the adapted TeamSTEPPS plus tailored implementation plan. In year 5 (not depicted), data analyses will be performed.

### Aim 1

We aim to characterize the use of urban school mental health services, including challenges and successes, using multiple sources of information (needs assessment, observation, and administrative data).

#### Procedure

##### Recruitment

Participants will be recruited from public and charter schools and agencies implementing and overseeing school mental health services in the local metro area. Participants for needs assessment interviews will range from upper-level leadership to front line personnel. Schools are invited to participate via an introductory email sent to district and/or school leadership. Following approval from the district and/or charter entities, individual staff members will be invited to participate via an introductory staff meeting or email facilitated by our leadership partners.

##### Needs Assessment

First, we will conduct a needs assessment with 15 key informants who represent clinical leadership, clinical and paraprofessional providers from the mental health team, teachers, and school administrators. Key informants will be asked about (1) the history of mental health services in their school/district, (2) successes, and (3) challenges or unintended consequences of previous models. We will ask interviewees to recommend additional key informants, and we will include as many individuals as appropriate to better our understanding of the district’s services. Key informant meetings will last approximately 1 hour.

##### Observations

We plan to observe school mental health teams providing services. Study staff will spend one full day per school observing team dynamics, service provision, and interactions with school personnel in 10 schools drawn from our partner districts. In light of COVID-19, if in-school observations are not permitted, we will utilize a “think aloud” exercise in lieu of observations.

##### Administrative Data

To understand the impact of previous and existing mental health service models in our partner districts, we will examine students’ behavioral health and school outcomes using administrative data when available (eg, Medicaid claims or school records, which vary by district). These data may include (1) routine and acute behavioral health service use, (2) psychotropic medication use, (3) school absence, (4) grade promotion, (5) school suspension, (6) disciplinary referrals, (7) individualized education plan status, and (8) demographic data including sex and race/ethnicity. School-level data will include average annual school-level absence and suspension rates, average annual number of students with disciplinary school transfers, and annual number of students with an individualized education plan.

#### Measures

We will use an adapted version of the Oxford Non-Technical Skills (NOTECHS) scale [39] as the field observation tool along with detailed qualitative field notes. The NOTECHS is a validated tool to assess teamwork and cognitive skills in the airline cockpit and has been reliably and validly modified for medical teams [40,41]. It measures leadership and management; teamwork and cooperation; problem solving and decision making; and situational awareness. Observers code team behavior in each domain using three to five items rated from 1 (below standard) to 4 (excellent). Subteam specific modifiers further examine the unique contributions of various staff roles. Previously, our team adapted and piloted the NOTECHS for school mental health teams [42].

#### Analysis Plan

##### Quantitative Analysis

One-way analysis of variance (ANOVA) will examine the mean differences between schools on the NOTECHS total and for each domain. We will synthesize previous local evaluations that provide insights into the characteristics of an urban setting school, including student demographics and school climate such as suspension and absence rates, as well as children’s behavioral health service use over time. The synthesis will inform key stakeholders about whether school mental health services have a positive effect on improving school outcomes and whether the school climate (school-level absence and suspension rates) moderates the effect of school mental health services on children’s school outcomes. We will share the results with key stakeholders and obtain their views on the successes and challenges of school-mental health service delivery.

##### Qualitative Analysis

We will load all field notes into NVivo (QSR International) for data management and analysis. Analysis will be guided by an integrated approach [43] that includes identification of a priori attributes of interest (ie, constructs from the CFIR and key TeamSTEPPS domains), combined with the identification of emergent codes and themes. This integrated approach uses an inductive process of iterative coding to identify recurrent themes, categories, and relationships. After initial data exploration, a comprehensive coding scheme is developed and applied to all data in order to produce a fine-grained descriptive analysis. A portion of the transcripts will be double coded to assess the reliability of the coding scheme. Disagreements in coding will be resolved through team discussion. Coders will be expected to reach and maintain reliability of κ ≥0.85.

##### Mixed Methods Analysis

We will integrate the NOTECHS observation data and field notes using the following taxonomy: the structure is Quan → Qual, the design is Convergent (we will use quantitative data [ie, NOTECHS] and qualitative data [ie, field notes] to explore similar questions to see if they reach the same conclusions), and the process is Connecting (to elaborate upon the quantitative findings to understand the process of implementation of school-based services as experienced by stakeholders) [44]. To integrate the quantitative and qualitative methods, we will follow the National Institutes of Health guidelines for best practices [45]. We will enter quantitative findings (ie, NOTECHS scores) into NVivo as attributes of each school. We will examine the distribution of NOTECHS scores and, if the distribution permits, will determine cut points to classify schools as high, medium, and low in team skills. Quantitative attributes will be used to categorize and compare important themes among subgroups and to triangulate to determine if quantitative and qualitative observational methods yield similar information.

### Aim 2

We aim to adapt TeamSTEPPS in collaboration with an advisory board of diverse stakeholders and develop tailored implementation strategies to support the use of TeamSTEPPS.

#### Procedure

Building on aim 1, we will establish an advisory board guided by the recommendations of Southam-Gerow et al [46] for utilizing stakeholder involvement in the treatment adaptation process. CFIR will guide the adaptation process [47]. Best practice recommendations for advisory boards will be followed, including establishing formalized commitment from members and clarifying expectations in advance [48]. The primary focus of the advisory board meetings will be to understand challenges, including, but not limited to, the problem of limited coordination and collaboration between mental health providers and school personnel, and to identify possible solutions and further TeamSTEPPS adaptations.

The advisory board will meet regularly to consider issues as the adaptation proceeds module by module. Consensus on important points will be determined by a 70% majority, consistent with the literature [48]. Meetings are expected to occur in multiple short sessions over the course of the school year (eg, 90 to 180-minute sessions every other month). However, community partner preferences will be accommodated for meeting time, length, and location, with options for virtual attendance (an entirely virtual advisory board may be necessitated by COVID-19). Participants will be compensated for advisory-board participation.

The products of aim 2 will be (1) an adapted TeamSTEPPS, directed toward both school mental health and school-employed personnel, and (2) specific tailored implementation strategies to improve services in schools in conjunction with TeamSTEPPS. Based on previous experience, we expect the implementation plan will include established strategies [49] such as providing teams with training and ongoing consultation, designating implementation champions in each school, and suggesting leaders implement policy mandates that address core components of TeamSTEPPS. For example, designing an asynchronous online training for new hires may be needed as our preliminary work indicated that staff turnover represents a barrier. The tailored implementation plan will be defined in the context of SISTER [36] strategies in accordance with the CFIR [49].

#### Recruitment

The precise schools and participants to include will be determined during aim 1 in collaboration with our district leadership partners. We anticipate 10 to 15 participants, which will likely include a variety of stakeholders, including school mental health providers, leadership from mental health organizations providing school-based services, teachers, school leaders/supervisors, and parents of youth receiving school-based mental health services. Participants will be formally invited by email or letter and asked to apply and agree in writing to participate to ensure they can commit for the duration of the research.

### Aim 3

We aim to explore the feasibility, acceptability, and utility of TeamSTEPPS plus the implementation strategies generated in aim 2 in terms of interprofessional collaboration, teamwork, and student outcomes.

#### Procedure

We will pilot test the adapted TeamSTEPPS and implementation strategies in eight schools. Participating mental health team members and school personnel will complete self-report assessments measuring interprofessional collaboration, teamwork, feasibility, and acceptability at baseline (ie, before engagement in TeamSTEPPS) and the school year following participation at two time points (Figure 2). Participants will be compensated for completion of self-report measures. We will obtain written informed consent from all participants.

#### Recruitment

District leadership will provide guidance on the schools to invite for participation in the TeamSTEPPS pilot. We will recruit schools randomly without replacement from the pool of potential schools. We will compare the eight schools who agree to participate with those who decline in order to explore representativeness (eg, compare on the size of the school and number of mental health staff in the school). We plan to enroll the first cohort of four schools during year 3 and the last cohort during year 4 of the study (Figure 2). We anticipate 10 participants per school consisting of mental health team staff, teachers, nonteaching staff, and at least one administrator (eg, principal), but will collect data from additional relevant personnel when possible determined in conjunction with principals.

#### Training in TeamSTEPPS

We will work with schools to ensure that the initial TeamSTEPPS training is provided during regular professional development time to reduce burden for staff. We expect the initial training to be about 4 hours and to be delivered in person or online. The exact plans for ongoing support will be informed by the implementation plan developed in aim 2 with the advisory board.

#### Measures

Feasibility and acceptability of the adapted TeamSTEPPS will be assessed using a combination of qualitative and quantitative methods. Exploratory outcomes include teamwork, interprofessional collaboration, and behavioral and academic outcomes for students receiving school-based services, as well as potential contextual predictors of implementation. Interprofessional collaboration will be assessed via observation and self-report.

#### Dependent Measures

The Acceptability of Intervention Measure (AIM) and Feasibility of Intervention Measure (FIM) are each reliable and valid four-item tools to assess perceptions of the acceptability and feasibility of TeamSTEPPS [50].

Mental health and school staff will complete the Expanded School Mental Health Collaboration Instrument-Community Version (ESMHCI-CV) [51]*,* a continuous measure of interprofessional collaboration in school mental health. Scores can be calculated individually or among groups working in the same school. For each subscale, an average score is calculated, with higher scores indicating strengths and lower scores indicating areas for improvement. The Cronbach α ranges from .81 to .94 for ESMHCI and .77 to .94 for ESMHCI-CV [51,52]

The TeamSTEPPS Teamwork Perceptions Questionnaire (T-TPQ) [53] is a 35-item self-report measure of individual perceptions of group-level team skills and behavior based on the five core components of teamwork that comprise TeamSTEPPS. Total scores are computed by summing all items, and higher scores indicate more favorable perceptions. The Cronbach α ranges from .88 to .95, and convergent validity is adequate [53].

The TeamSTEPPS Teamwork Attitudes Questionnaire (T-TAQ) [54] is a 30-item self-report measure of individual attitudes related to the core TeamSTEPPS components. A sum score is calculated across items, with higher scores indicating more positive attitudes. Constructs exhibit unique variance, and the Cronbach α ranges from .70 to .83 [54].

#### Exploratory Contextual Predictors

The Evidence-Based Practice Attitude Scale (EBPAS) [55] is a 15-item self-report measure of attitudes toward adoption of evidence-based practice (EBP). It consists of the following four subscales: appeal (is EBP intuitively appealing), requirements (would an EBP be used if required), openness (general openness to innovation), and divergence (perceived divergence between EBP and current practice). Higher scores indicate more positive attitudes, with the exception of divergence, which is reverse coded. The EBPAS has national norms, demonstrated validity, and good internal consistency (subscale α range from .67 to .91) [56,57].

The Maslach Burnout Inventory Human Services Survey (MBI) [58] is a 22-item self-report measure of burnout. Three subscales measure emotional exhaustion, depersonalization, and reduced personal accomplishment. Items are rated from 0 (never) to 6 (everyday), with higher scores on emotional exhaustion and depersonalization, and lower scores on personal accomplishment (reverse scored) indicating higher levels of burnout. Satisfactory internal consistency, and discriminant and factorial validity have been demonstrated [59-61].

#### Observations

Consistent with aim 1, a trained observer will spend one full day taking detailed field notes on team dynamics, service provision, and interactions among mental health providers, school personnel, and students along with using the adapted NOTECHS.

#### Interviews

We anticipate conducting 12 individual interviews of people randomly selected from all of the different stakeholder groups engaged in TeamSTEPPS, but will continue until saturation is achieved [62]. The semistructured interview protocol will ensure uniform inclusion and sequencing of topics and allow for valid comparison across interviews. The interview guide will have three parts, with the first and second querying about feasibility and acceptability, respectively. In the third section, we will present findings from the quantitative data assessing interprofessional collaboration and student outcomes and ask for respondent’s reflections.

#### Administrative Data

In districts where required data are available, behavioral and educational outcomes of students will be analyzed using deidentified administrative data as described in aim 1.

#### Analysis Plan

##### Qualitative Analysis

We will use NVivo for analysis using the integrated approach as described in aim 1.

##### Quantitative Analysis

Feasibility will be determined by the proportion of schools that enroll/those that are invited and the proportion of participants who attend TeamSTEPPS training/those who are eligible to participate. We will also examine the distribution of AIM and FIM scores.

Collaboration and teamwork will be assessed observationally (ie, NOTECHS) and via self-report (eg, ESMHCI and T-TPQ). The mean total and domain scores in the NOTECHS will be computed for each school. Individual and school scores will be calculated for self-report measures. A series of one-way ANOVA assessments will examine differences between schools at each time point to assess the impact of TeamSTEPPS plus implementation strategies. Repeated measures ANOVA will examine scores on these measures within schools over time. Relevant individual and team factors, including EBPAS and MBI scores and team size, will be explored and included in the models as covariates where appropriate.

Behavioral and academic outcomes will be explored using administrative data as described in aim 1. These methods are described in detail elsewhere [63-67]. Using repeated measures ANOVA, we will compare student outcomes for students receiving mental health services the one full school year prior to the school’s engagement in TeamSTEPPS in the subsequent school year. When data are available, we will explore whether school absence, suspension, and grade promotion differ 1 year before and after the implementation at the individual and school levels. Additionally, logistic regression analyses will examine if children’s behavioral health service use differs before and after the school’s engagement in TeamSTEPPS. All analyses conducted will accommodate nesting of participants (ie, teachers and mental health professionals) within clusters (ie, schools).

##### Mixed Methods Analysis

We will integrate the NOTECHS*,* self-report, and administrative data with the interviews and field notes following best practices [44]. The structure is Quan → Qual, the function is Complementarity, and the process is Connecting [43]. We will use findings from the quantitative data to identify patterns in the qualitative data. To do this, we will enter quantitative findings (eg NOTECHS ratings) into NVivo as attributes of each (1) school (for field notes) and (2) individual (for interviews).

## Results

Recruitment for aim 1 of the study has begun. Goals for aim 1 are expected to be completed in Spring 2021. This project has been reviewed by the University of Pennsylvania (protocol 834488) and City of Philadelphia Institutional Review Board (study #2020-31).

## Discussion

This study will examine the acceptability and feasibility of an adapted team training intervention, TeamSTEPPS, within school mental health. Our previous trial demonstrated both the preliminary acceptability and challenges of implementing TeamSTEPPS with school mental health service providers [33,34]. Results from the initial trial indicated the need to gather additional stakeholder feedback about TeamSTEPPS, the potential benefit of a co-developed tailored implementation plan, and the importance of leadership support.

This study has three main innovations. First, few implementation studies focus on improving interorganizational functioning [68]. Specific rigorous strategies to facilitate the alignment of two distinct yet related service settings, such as schools and contracted mental health teams, have not been sufficiently explored, contributing to the importance of this study. Second, we will use a community-partnered approach to engage stakeholders in understanding school mental health collaboration challenges and identifying solutions [46]. We will triangulate stakeholder perspectives, observations, and administrative data, moving the field beyond traditional trial and error implementation and improving rigor in implementation science. Third, few studies on school mental health services have given an equal voice to stakeholders from both schools and community mental health. TeamSTEPPS provides structured processes for improving collaboration across a range of stakeholders. While there are some limitations, including the lack of a randomized control group for aim 3, this project has the potential to improve school culture and climate, which in turn may improve student outcomes [32] and ready both mental health and school systems for EBP implementation. We anticipate that this project will lead to future studies testing the adapted TeamSTEPPS plus tailored implementation strategies in a randomized multisite implementation trial. The ultimate goal is to improve the quality of services underserved children receive in schools.

## Appendix

Multimedia Appendix 1 Peer-review report from the Agency for Healthcare Research and Quality.

## Abbreviations

AIM: Acceptability of Intervention Measure
ANOVA: analysis of variance
CFIR: Consolidated Framework for Implementation Research
EBP: evidence-based practice
EBPAS: Evidence-Based Practice Attitude Scale
ERIC: Expert Recommendations for Implementing Change
ESMHCI: Expanded School Mental Health Collaboration Instrument
FIM: Feasibility of Intervention Measure
MBI: Maslach Burnout Inventory Human Services Survey
NOTECHS: Oxford Non-Technical Skills
SISTER: School Implementation Strategies, Translating Expert Recommendations for Implementing Change Resources
TeamSTEPPS: Team Strategies and Tools to Enhance Performance and Patient Safety
T-TAQ: Teamwork Attitudes Questionnaire
T-TPQ: TeamSTEPPS Teamwork Perceptions Questionnaire

## References

[1] Cree RA, Bitsko RH, Robinson LR, Holbrook JR, Danielson ML, Smith C, Kaminski JW, Kenney MK, Peacock G (2018). Health Care, Family, and Community Factors Associated with Mental, Behavioral, and Developmental Disorders and Poverty Among Children Aged 2–8 Years — United States, 2016. MMWR and Morbidity and Mortality Weekly Report.

[2] Tolan PH, Henry D (1996). Patterns of psychopathology among urban poor children: Comorbidity and aggression effects. Journal of Consulting and Clinical Psychology.

[3] Acri MC, Bornheimer LA, O'Brien K, Sezer S, Little V, Cleek AF, McKay MM (2016). A model of integrated health care in a poverty-impacted community in New York City: Importance of early detection and addressing potential barriers to intervention implementation. Soc Work Health Care.

[4] Merikangas KR, He JP, Brody D, Fisher PW, Bourdon K, Koretz DS (2010). Prevalence and treatment of mental disorders among US children in the 2001-2004 NHANES. Pediatrics.

[5] Kataoka SH, Zhang L, Wells KB (2002). Unmet need for mental health care among U.S. children: variation by ethnicity and insurance status. Am J Psychiatry.

[6] Pumariega AJ, Rogers K, Rothe E (2005). Culturally competent systems of care for children's mental health: advances and challenges. Community Ment Health J.

[7] Cummings JR, Ponce NA, Mays VM (2010). Comparing racial/ethnic differences in mental health service use among high-need subpopulations across clinical and school-based settings. J Adolesc Health.

[8] Wang K, Chen Y, Zhang J, Oudekerk B (2020). Indicators of School Crime and Safety: 2019. National Center for Education Statistics.

[9] US Department of Health and Human Services, US Department of Education, US Department of Justice (2000). Report of the Surgeon General's Conference on Children's Mental Health: A National Action Agenda.

[10] Atkins MS, Shernoff ES, Frazier SL, Schoenwald SK, Cappella E, Marinez-Lora A, Mehta TG, Lakind D, Cua G, Bhaumik R, Bhaumik D (2015). Redesigning community mental health services for urban children: Supporting schooling to promote mental health. J Consult Clin Psychol.

[11] Foster S, Rollefson M, Doksum T, Noonan D, Robinson G, Teich J (2005). School Mental Health Services in the United States, 2002-2003.

[12] Bates S, Mellin E, Paluta L, Anderson-Butcher D, Vogeler M, Sterling K (2019). Examining the Influence of Interprofessional Team Collaboration on Student-Level Outcomes through School–Community Partnerships. Children & Schools.

[13] Ball A, Anderson-Butcher D, Mellin EA, Green JH (2010). A Cross-Walk of Professional Competencies Involved in Expanded School Mental Health: An Exploratory Study. School Mental Health.

[14] Choi BCK, Pak AWP (2007). Multidisciplinarity, interdisciplinarity, and transdisciplinarity in health research, services, education and policy: 2. Promotors, barriers, and strategies of enhancement. Clin Invest Med.

[15] Hall P (2005). Interprofessional teamwork: professional cultures as barriers. J Interprof Care.

[16] Holmesland A, Seikkula J, Nilsen O, Hopfenbeck M, Erik Arnkil T (2010). Open Dialogues in social networks: professional identity and transdisciplinary collaboration. Int J Integr Care.

[17] Lynn CJ, McKay MM, Atkins MS (2003). School Social Work: Meeting the Mental Health Needs of Students through Collaboration with Teachers. Children & Schools.

[18] Ødegård A (2005). Perceptions of interprofessional collaboration in relation to children with mental health problems. A pilot study. J Interprof Care.

[19] Ekornes S (2015). Teacher Perspectives on Their Role and the Challenges of Inter-professional Collaboration in Mental Health Promotion. School Mental Health.

[20] Franklin CG, Kim JS, Ryan TN, Kelly MS, Montgomery KL (2012). Teacher involvement in school mental health interventions: A systematic review. Children and Youth Services Review.

[21] Gregory M, Feitosa J, Driskell T, Salas E, Vessey W, Salas E, Tannenbum S, Cohen D, Latham G (2013). Designing, Delivering, and Evaluating Team Training in Organizations: Principles That Work. Developing and Enhancing Teamwork in Organizations: Evidence-based Best Practices and Guidelines.

[22] Wagner E, Austin B, Davis C, Hindmarsh M, Schaefer J, Bonomi A (2001). Improving chronic illness care: translating evidence into action. Health Aff (Millwood).

[23] King H, Battles J, Baker D, Alonso A, Salas E, Webster J, Toomey L, Salisbury M (2008). TeamSTEPPS™: Team Strategies and Tools to Enhance Performance and Patient Safety. Advances in Patient Safety: New Directions and Alternative Approaches (Vol. 3: Performance and Tools).

[24] TeamSTEPPS. Agency for Healthcare Research and Quality.

[25] TeamSTEPPS® Instructor Guide. Agency for Healthcare Research and Quality.

[26] Mahoney J, Ellis T, Garland G, Palyo N, Greene P (2012). Supporting a psychiatric hospital culture of safety. J Am Psychiatr Nurses Assoc.

[27] Mayer C, Cluff L, Lin W, Willis T, Stafford R, Williams C, Saunders R, Short KA, Lenfestey N, Kane HL, Amoozegar JB (2011). Evaluating Efforts to Optimize TeamSTEPPS Implementation in Surgical and Pediatric Intensive Care Units. The Joint Commission Journal on Quality and Patient Safety.

[28] Sheppard F, Williams M, Klein V (2013). TeamSTEPPS and patient safety in healthcare. J Healthc Risk Manag.

[29] Van Bogaert P, Timmermans O, Weeks S, van Heusden D, Wouters K, Franck E (2014). Nursing unit teams matter: Impact of unit-level nurse practice environment, nurse work characteristics, and burnout on nurse reported job outcomes, and quality of care, and patient adverse events--a cross-sectional survey. Int J Nurs Stud.

[30] Vertino K (2014). Evaluation of a TeamSTEPPS© Initiative on Staff Attitudes Toward Teamwork. JONA: The Journal of Nursing Administration.

[31] Stead K, Kumar S, Schultz T, Tiver S, Pirone C, Adams R, Wareham CA (2009). Teams communicating through STEPPS. Med J Aust.

[32] Caldarella P, Shatzer R, Gray K, Young K, Young E (2015). The Effects of School-wide Positive Behavior Support on Middle School Climate and Student Outcomes. RMLE Online.

[33] Wolk C, Stewart R, Cronholm P, Eiraldi R, Salas E, Mandell D (2019). Adapting TeamSTEPPS for school mental health teams: a pilot study. Pilot Feasibility Stud.

[34] Wolk C, Stewart R, Eiraldi R, Cronholm P, Salas E, Mandell D (2019). The implementation of a team training intervention for school mental health: Lessons learned. Psychotherapy (Chic).

[35] Damschroder L, Aron D, Keith R, Kirsh S, Alexander J, Lowery J (2009). Fostering implementation of health services research findings into practice: a consolidated framework for advancing implementation science. Implement Sci.

[36] Cook C, Lyon A, Locke J, Waltz T, Powell B (2019). Adapting a Compilation of Implementation Strategies to Advance School-Based Implementation Research and Practice. Prev Sci.

[37] Proctor E, Silmere H, Raghavan R, Hovmand P, Aarons G, Bunger A, Griffey R, Hensley M (2011). Outcomes for implementation research: conceptual distinctions, measurement challenges, and research agenda. Adm Policy Ment Health.

[38] Brown C, Curran G, Palinkas L, Aarons G, Wells K, Jones L, Collins LM, Duan N, Mittman BS, Wallace A, Tabak RG, Ducharme L, Chambers DA, Neta G, Wiley T, Landsverk J, Cheung K, Cruden G (2017). An Overview of Research and Evaluation Designs for Dissemination and Implementation. Annu Rev Public Health.

[39] Flin R, Martin L, Goeters K, Hormann H, Amalberti R, Valot C (2003). Development of the NOTECHS (non-technical skills) system for assessing pilots' CRM skills. Human Factors and Aerospace Safety.

[40] Mishra A, Catchpole K, McCulloch P (2009). The Oxford NOTECHS System: reliability and validity of a tool for measuring teamwork behaviour in the operating theatre. Qual Saf Health Care.

[41] Catchpole K, Giddings A, Hirst G, Dale T, Peek G, de Leval MR (2007). A method for measuring threats and errors in surgery. Cogn Tech Work.

[42] Wolk CB, Mandell DM (2016). Observing teamwork in school mental health: The adaptation and refinement of the Oxford NOTECHS System.

[43] Bradley E, Curry L, Devers K (2007). Qualitative data analysis for health services research: developing taxonomy, themes, and theory. Health Serv Res.

[44] Palinkas L, Aarons G, Horwitz S, Chamberlain P, Hurlburt M, Landsverk J (2011). Mixed method designs in implementation research. Adm Policy Ment Health.

[45] NIH Office of Behavioral and Social Sciences Research (2018). Best Practices for Mixed Methods Research in the Health Sciences.

[46] Southam-Gerow M, Hourigan S, Allin R (2009). Adapting evidence-based mental health treatments in community settings: preliminary results from a partnership approach. Behav Modif.

[47] Damschroder L, Peikes D, Petersen D (2013). Using Implementation Research to Guide Adaptation, Implementation, and Dissemination of Patient-Centered Medical Home Models. Agency for Healthcare Research and Quality.

[48] Newman S, Andrews J, Magwood G, Jenkins C, Cox M, Williamson D (2011). Community advisory boards in community-based participatory research: a synthesis of best processes. Prev Chronic Dis.

[49] Powell B, Waltz T, Chinman M, Damschroder L, Smith J, Matthieu M, Proctor EK, Kirchner JE (2015). A refined compilation of implementation strategies: results from the Expert Recommendations for Implementing Change (ERIC) project. Implement Sci.

[50] Weiner B, Lewis C, Stanick C, Powell B, Dorsey C, Clary A, Boynton MH, Halko H (2017). Psychometric assessment of three newly developed implementation outcome measures. Implement Sci.

[51] Mellin E, Taylor L, Weist M, Lockhart N (2015). The Expanded School Mental Health Collaboration Instrument [Community Version]: Development and Initial Psychometrics. School Mental Health.

[52] Mellin E, Taylor L, Weist M (2013). The Expanded School Mental Health Collaboration Instrument [School Version]: Development and Initial Psychometrics. School Mental Health.

[53] (2010). TeamSTEPPS® Teamwork Perceptions Questionnaire (T-TPQ) Manual. Agency for Healthcare Research and Quality.

[54] Baker D, Krokos K, Amodeo A (2017). TeamSTEPPS® Teamwork Attitudes Questionnaire Manual. Agency for Healthcare Research and Quality.

[55] Aarons G (2004). Mental Health Provider Attitudes Toward Adoption of Evidence-Based Practice: The Evidence-Based Practice Attitude Scale (EBPAS). Ment Health Serv Res.

[56] Aarons G, Glisson C, Hoagwood K, Kelleher K, Landsverk J, Cafri G (2010). "Psychometric properties and U.S. national norms of the Evidence-Based Practice Attitude Scale (EBPAS)": Correction to Aarons et al. (2010). Psychological Assessment.

[57] Aarons G, Sawitzky A (2006). Organizational Culture and Climate and Mental Health Provider Attitudes Toward Evidence-Based Practice. Psychol Serv.

[58] Maslach C, Jackson S, Leiter M (1996). Maslach Burnout Inventory Manual.

[59] Maslach C, Schaufeli W, Leiter M (2001). Job burnout. Annu Rev Psychol.

[60] Schaufeli W, Bakker A, Hoogduin K, Schaap C, Kladler A (2001). on the clinical validity of the maslach burnout inventory and the burnout measure. Psychol Health.

[61] Glass D, McKnight JD (1996). Perceived control, depressive symptomatology, and professional burnout: A review of the evidence. Psychology & Health.

[62] Guest G, Bunce A, Johnson L (2016). How Many Interviews Are Enough?. Field Methods.

[63] Kang-Yi Christina D, Locke Jill, Pellecchia Melanie, Marcus Steve C, Hadley Trevor, Mansell David S (2016). Decline in Medicaid-Funded One-to-One Behavioral Support Use in School as Children Age. School Ment Health.

[64] Kang-Yi Christina D, Locke Jill, Pellecchia Melanie, Marcus Steve C, Hadley Trevor, Mandell Ds (2016). Erratum to: Decline in Medicaid-Funded One-to-One Behavioral Support Use in School as Children Age. School Mental Health.

[65] Kang-Yi Christina D, Wolk Courtney Benjamin, Locke Jill, Beidas Rinad S, Lareef Ishara, Pisciella Aelesia E, Lim Suet, Evans Arthur C, Mandell David S (2018). Impact of school-based and out-of-school mental health services on reducing school absence and school suspension among children with psychiatric disorders. Eval Program Plann.

[66] Kang-Yi Christina D, Locke Jill, Marcus Steven C, Hadley Trevor R, Mandell David S (2016). School-Based Behavioral Health Service Use and Expenditures for Children With Autism and Children With Other Disorders. Psychiatr Serv.

[67] Kang-Yi Christina D, Mandell David S, Hadley Trevor (2013). School-based mental health program evaluation: children's school outcomes and acute mental health service use. J Sch Health.

[68] Stark K, Arora P, Funk C (2011). Training school psychologists to conduct evidence-based treatments for depression. Psychol. Schs.

