# Supplementary material for: Team Strategies and Tools to Enhance Performance and Patient Safety (TeamSTEPPS) to Improve Collaboration in School Mental Health: Protocol for a Mixed Methods Hybrid Effectiveness-Implementation Study
Source: JMIR Res Protoc. 2021 Feb 8;10(2):e26567. doi: 10.2196/26567 (PMC7899798; doi:10.2196/26567)
Supplement: Multimedia Appendix 1 [file resprot_v10i2e26567_app1.pdf]

SUMMARY STATEMENT

(Privileged Communication)

PROGRAM CONTACT:
Monika Haugstetter
301-427-1515
monika.haugstetter@ahrq.hhs.gov

Release Date: **10/22/2019**
Revised Date:

Application Number: **1 R18 HS026862-01A1**

Principal Investigator

WOLK, COURTNEY LYNN BENJAMIN

Applicant Organization: UNIVERSITY OF PENNSYLVANIA

Review Group: **HSQR**
Healthcare Patient Safety and Quality Improvement Research

Meeting Date: **10/10/2019**
Council: **JAN 2020**
Requested Start: **04/01/2020**

RFA/PA: **PA18-793**
PCC: **CQUIPS**

Project Title: **Adapting and implementing TeamSTEPPS in school mental health**

SRG Action: [redacted] [redacted]

Human Subjects: 30-Human subjects involved - Certified, no SRG concerns
Animal Subjects: 10-No live vertebrate animals involved for competing appl.
Gender: 1A-Both genders, scientifically acceptable
Minority: 1A-Minorities and non-minorities, scientifically acceptable
Clinical Research - not NIH-defined Phase III Trial

[redacted]

ADMINISTRATIVE BUDGET NOTE: The budget shown is the requested budget and has not been adjusted to reflect any recommendations made by reviewers. If an award is planned, the costs will be calculated by Institute grants management staff based on the recommendations outlined below in the COMMITTEE BUDGET RECOMMENDATIONS section.

RESUME AND SUMMARY OF DISCUSSION:
This resubmission application titled, *Adapting and implementing TeamSTEPPS in school mental health* by Dr. Courtney Wolk of the University of Pennsylvania responds to PA18-793: AHRQ Health Services Research Demonstration and Dissemination Grants (R18). It proposes to “collaboratively identify solutions to challenges in collocated school-based mental health services based upon the feedback of stakeholders using TeamSTEPPS to support mental health team-school collaboration.” The application has various strengths. Preliminary work with school-based mental health teams in Philadelphia suggested a lack of integration and alignment of mental health services with the educational mission, agenda and priorities. The team with multiple expertise include implementation science, team science, school mental health research, and relevant analytic approaches. The co-investigators are accomplished researchers with proven track record in grants and publications. They plan to refine and adapt an already known intervention to a new setting and to examine broader contextual organizational processes and stakeholder involvements in school setting. The scales to assess TeamSTEPPS outcomes are well validated. The mixed methods approach are well outlined and developed for general medical settings to a collocated mental health in schools setting. The outcomes focus appropriately on feasibility and acceptability but they do include behavioral and educational outcomes. The environment is supportive. Reviewers have also noted some weaknesses. There is no review of national experience with school-based mental health care. It is unclear why the previous experiences school based mental health have failed and what pitfalls the present proposal needs to address to avoid similar outcome. The PI is relatively junior investigator. The team remains investigator-heavy with overlap of expertise. No assessment of parents’ opinion of outcome has been outlined. There are not enough teachers, students and family members interviewed. The instrument used to assess study outcome of interprofessional collaboration is still not well validated. The link between failure of previous mental health service models and the proposed model as a remedial model is not clear and not supported by the data presented. The time-line (5 years) seems too excessive. Overall, the reviewers have rated this application from “Excellent” to “Outstanding” with a High level of enthusiasm.

DESCRIPTION (provided by applicant): Children obtain more mental health services through schools than through any other public system or agency. School-based mental health care often is provided by community mental health clinicians contracted to deliver school-based services. A rate-limiting step to the team's success in providing effective, evidence-based care is that the mission and work of the mental health teams, whose focus is on behavioral health service delivery, is not always consistent with the educational mission and needs of under-resourced schools. Participants will be drawn from school-based teams in Philadelphia's community mental health network and public schools. Existing methods to enhance the adoption, implementation, and sustainability of evidence-based practices have not addressed the challenges that this common model of collocated services poses, such as disparate goals and lack of coordination and collaboration. Team Strategies and Tools to Enhance Performance and Patient Safety (TeamSTEPPS) has been widely disseminated in health care settings and provides a promising strategy for addressing communication and climate in school mental health teams. Philadelphia provides an ideal context in which to explore the impact of TeamSTEPPS on inter-organizational processes because Philadelphia relies heavily on mental health agencies to provide services in schools. Most students are low income minority children, allowing us to simultaneously address multiple AHRQ priority populations. We propose to take a participatory approach to collaboratively identify solutions to challenges in collocated school- based mental health services based upon the feedback of stakeholders using TeamSTEPPS to support mental health team-school collaboration. Consistent with AHRQ priorities to minimize adverse events, maximize recovery and support 360° person-centered team-based care, we propose the following aims. First, in Aim 1 we will capture key stakeholder perspectives about challenges in collocated school mental health services through formative work to inform collaborative planning and capacity building activities in Aim 2. Then, in Aim 2 we will identify inter-organizational challenges and required components of TeamSTEPPS to adapt. We will establish an advisory board and adapt TeamSTEPPS. The product of Aim 2 will be an adapted TeamSTEPPS, directed toward both school mental health and school-employed personnel, and specific, tailored implementation strategies to improve services in schools in conjunction with

TeamSTEPPS. Finally, we will explore the feasibility, acceptability, and utility of TeamSTEPPS and the strategies generated in Aim 2 on inter- professional collaboration, teamwork, and student outcomes in eight schools.

PUBLIC HEALTH RELEVANCE: The present study has the potential to impact public health by improving the quality of mental health services received by children in schools. The research activities will improve our understanding of the complex problems that may occur when mental health services are collocated in schools and examine possible solutions. Intervention and implementation strategies to support this type of work will be important going forward given that s c hool dis tric ts are inc reas ingly relying on c ontrac ted m ental health providers and c olloc ation is a c om m on m odel in other settings (e.g., primary care).

CRITIQUES: The written critiques of individual reviewers are provided in essentially unedited form below. These critiques were prepared prior to the meeting and may not have been revised afterwards. The “Resume and Summary of Discussion” above summarizes the final opinions of the committee.

CRITIQUE 1

[redacted]		[redacted]	
[redacted]		[redacted]	
[redacted]		[redacted]	
[redacted]		[redacted]	
[redacted]		[redacted]	

Overall Impact:

Strengths

- This is a revised application that aims at adapting TeamSTEPPS to support mental health team-school collaboration. The study aims at remediating the challenge posed by the lack of shared vision and mission of behavioral mental health service delivery in school and its educational mission. This diversion in mission forms the rate-limiting step in providing evidence-based mental health. The investigators preliminary work with school-based mental health teams in Philadelphia suggested lack of integration and alignment of mental health services with the educational mission, agenda and priorities.
- This study addresses a significant challenge which is offering effective metal health services in school. Multiple previous models have been attempted but apparently failed (based on personal communication to the investigators). The current proposal will adapt a model that have demonstrated utility in other settings.
- The investigative team is very experienced in this proposed model and have completed preliminary work within school and this application built on their previous experience which identified major challenges as communication and role definition.
- Study’s first aim is to capture key stakeholder perspectives about challenges in implementing mental health services in school and conduct a needs assessment with 15 key informants to understand why previous models of school-based mental health services have not improved child outcomes including inter-organizational challenges.
- Aim 2 will focus on identification of inter-organizational challenges and required components of TeamSTEPPS to adapt, which will be accomplished through the advisory board consultative process. Stakeholders will include school mental health and school-employed personnel.
- Aim 3 will involve an implementation feasibility, acceptability and utility of the optimized TeamSTEPPS,

Weaknesses

- Reportedly multiple evidence based models have failed to enhance mental health care within the school system. References to that failure is based on personal communication only. It

was unclear why the previous experiences have failed and what pitfalls the present proposal needs to address to avoid similar outcome. While this will be the goal of one study aim, it would have been helpful to have working hypotheses about the reasons for failure based on preliminary discussions with key informants from those experiences.

1. Significance:
Strengths
- This study addresses a significant problem which is providing effective mental health services in school.
- Providing mental health service within the school is an important goal and could be particularly impacting for children from low income and disadvantaged household.
- The proposed model is designed to address two identified challenges for a successful integration of such services. More organic integration of mental health services within the educational goals and organizational structure is proposed to overcome some of the identified challenges.
- If successful, this study may provide a successful model replicable in similar setting nationwide.

Weaknesses
- Limited evidence is presented to justify that the presented model will address the barriers to a better implementation of mental health services in school.

2. Investigators:
Strengths
- Team with multiple expertise include implementation science, team science, school mental health research, and the relevant analytic approaches.
- Dr. Wolk, PhD (PI) is an Assistant Professor of Psychiatry at the Perelman School of Medicine, University of Pennsylvania and Director of Clinical Training at the Center for Mental Health. She is an implementation science researcher focusing on the implementation of evidence-based practices in mental health in school, community, and healthcare settings and she recently completed a focused F32 award. She is experienced in implementation science.
- The investigative team include Kamilah Jackson, MD with main role of community liaison partner, Ricardo Eiraldi, PhD (Co-I) Associate Professor of Clinical Psychology in Pediatrics at Children's Hospital of Philadelphia and the Division of Developmental and Behavioral Pediatrics and Department of Psychiatry, University of Pennsylvania and experienced in dissemination implementation science, Steven C. Marcus, PhD (Co-I) is Associate Professor at the University of Pennsylvania School of Social Policy and Practice and very experienced statistician and epidemiologist, Christina Kang-Yi, PhD (Co-I) is a Research Assistant Professor in the Department of Psychiatry, Perelman School of Medicine at the University of Pennsylvania, experienced in publicly funded program evaluations, Eduardo Salas, PhD (Consultant) is Professor and Allyn R. & Gladys M. Cline Department Chair in the Department of Psychological Sciences at Rice University. He is a senior scientist who will consult on team science, Ming Xie highly experienced data analyst working with Medicaid and other health claims data.

Weaknesses
- PI relatively junior investigator
- Still too many investigators

3. Innovation:
Strengths
- This study is moderately innovative in terms of refining and adapting an already known intervention to a new setting and in examining broader contextual organizational processes and stakeholder involvements in school setting.
- Use of nomenclature emphasizing student or children instead of patients

Weaknesses
- None noted.

4. Approach:
Strengths

- Preliminary work indicated TeamSTEPPS was feasible and acceptable to implement.
- Identified leadership as an important facilitator.
- Identified barriers included staff turnover, lack of resources, and challenges in the school-mental health team relationship
- Using the Consolidated Framework for Implementation Research (CFIR) Implementation Model as an overarching framework.
- The approach has 3 aims: An interview with stakeholders to understand why previous model failed, the formation of an advisory board to develop an adapted model to apply to the school and then a feasibility and acceptability trials.
- Assessment of outcome will include collaboration and teamwork (via self-report and observation), and of students' behavioral and educational outcomes.
- TeamSTEPPS has been adapted to multiple health care setting
- Aims 1 will include 3 main activities: An interview with key stake holders regarding previous failed experiences, an observation of 5 successful MH teams and 5 underperforming MH team and team work will be assesses using the Oxford Non-Technical Skills (NOTECHS) scale, the third activity is to examine students' behavioral health and school outcomes, by analyzing Medicaid claims data.
- Widely representative group of stakeholders including school mental health providers (including clinicians and paraprofessionals), teachers, school and agency leaders/supervisors, parents of youth receiving school-based mental health services, and stakeholders from [redacted].
- Aim 3 will test the feasibility and acceptability on inter-professional collaboration, teamwork, and student outcomes in eight schools.
- Scales to assess TeamSTEPPS outcomes are well validated.

Weaknesses

- There is no review of national experience with school-based mental health care.
- Time-line (5 years) seems too excessive, for example an entire year is allowed for the advisory group to formulate the adapted model.
- The integration of the data gathered from aim 1 and how it will help to adapt the proposed model was not clear as the role of the Medicaid claim data.
- The advisory board in aim 2 has an a priori primary focus which is stated as to understand challenges, including but not limited to the problem of limited coordination and collaboration between mental health providers and school personnel, and identify possible solutions and further TeamSTEPPS adaptations. If these issues have been identified a priori, it was unclear whether the insight derived from aim 1 will address these issues and how data derived from aim 1 will inform aim 2.
- The process of aim 2 in terms of themes is not very well specified and open ended.
- Administrative data is collected during 1st year and apparently the year after the intervention, however the table of the study timeline is confusing and does not reflect this schedule.
- No assessment of parents' opinion of outcome.
- Instrument used to assess study outcome of interprofessional collaboration is still not well validated.
- The link between failure of previous mental health service models and the proposed model as a remedial model is not clear and not supported by the data presented.
- The interview assessing the feasibility and acceptability of TeamSTEPPS has not been developed yet.
- Evaluation of the potential dissemination of the model to other schools seems premature if the model's utility has not been proven yet.
- Nebulous calculation of sample size needed.

5. Environment:
Strengths
- Very supportive environment, including letter of support from School Superintendent.

Weaknesses
- None noted.

Protections for Human Subjects: Acceptable

Degree of Responsiveness: Responsive

Inclusion of Women and Minority Subjects: Acceptable

Inclusion of AHRQ Priority Populations: Acceptable

Budget and Period of Support: Appropriate

Authentication of Key Resources Used:

Resubmission Applications:
Strengths
- Resubmission was responsive to previous review although issues remain in the approach and it is unclear whether the proposed model will adequately addresses the challenges faced by previous mental health implementation attempts.

Weaknesses
- None noted.

CRITIQUE 2

[redacted]		[redacted]	
[redacted]		[redacted]	
[redacted]		[redacted]	
[redacted]		[redacted]	
[redacted]		[redacted]	

Overall Impact:
This is a resubmission. [redacted]
Strengths
- Innovative application of a proven organizational change intervention
- Strong investigators

Weaknesses
- Investigator-heavy team
- Mentions a future R01 several times, but does not articulate it in future directions

1. Significance:
Strengths
- Mental illness among disadvantaged children is a significant problem.
- These children typically get mental healthcare in their schools. However, the coordination between mental health professionals and school education professionals do not necessarily work smoothly.
- Thus, an intervention to coordinate an effective communication and collaboration between these 2 professional groups would be a significant benefit.

Weaknesses

- Previous critique raised doubt in significance, stating that TeamSTEPPS is well studied in health care settings, and is already known to improve the function of teams in organizational structures. However, no evidence-based program has succeeded in improving outcomes of students with mental health concerns in the [redacted] schools over the past 10 years. So, it would seem logical to implement a proven tool in this challenging environment.

2. Investigators:

Strengths

- The PI, Dr. Wolk, while having her terminal degree fairly recently in 2012, already has 38 publications and has established herself as an implementation scientist specializing in mental health issues.
- The co-investigators are very accomplished researchers with proven track record in grants and publications in relevant areas. There is appropriate community partnership.

Weaknesses

- The team remains investigator-heavy with overlap of expertise despite previous critique.

3. Innovation:

Strengths

- Focus on improving inter-organizational functioning
- Community-partnered approach
- Equal voice to stakeholders from both school and community mental health

Weaknesses

- Previous critique addressed that TeamSTEPPS has already shown effectiveness in various settings, especially healthcare. However, it has not been specifically applied in school-mental health interface settings, and the lack of effectiveness of 5 previous evidence-based interventions over the past 10 years would actually increase the importance of implementing this intervention in this challenging environment.

4. Approach:

Strengths

- Appropriate use of Consolidated Framework for Implementation Research (CFIR) as the overarching framework
- TeamSTEPPS was developed by the U.S. Department of Defense Patient Safety Program in collaboration with AHRQ. It is designed to build competencies in the areas of leadership, situation monitoring, mutual support, and communication.
- The PI has already adapted and piloted TeamSTEPPS for use with school mental health teams.
- Study protocol will be published in Year 1 to ensure rigor and transparency.
- Appropriate representation in advisory boards that is responsive to the previous critique
- Well outlined mixed methods approach that has responded to previous critiques

Weaknesses

- Might want to distribute the time allocation of the Aims differently, a bit shorter on Aim 2.

5. Environment:

Strengths

- University of Pennsylvania is an outstanding institution.
- Established working relationship with schools and mental health professionals

Weaknesses

- None

Protections for Human Subjects: Acceptable

Degree of Responsiveness: Acceptable

Inclusion of Women and Minority Subjects: Acceptable

Inclusion of AHRQ Priority Populations: Acceptable

Budget and Period of Support: Acceptable

Authentication of Key Resources Used: N/A

Resubmission Applications: Responsive to the previous critiques

CRITIQUE 3

[redacted]		[redacted]	
[redacted]		[redacted]	
[redacted]		[redacted]	
[redacted]		[redacted]	
[redacted]		[redacted]	

Overall Impact:

Strengths

- This is an ambitious and innovative project that aims to implement Team STEPPS to address access to high quality mental health care within public school systems in an urban setting.
- The team has developed TeamSTEPPS to address mental health care team dynamics and now want to do interorganizational development to address collocated school services.
- The investigators commit to publishing the study protocol in Year 1 and publish white papers of their Center's website. They imply there will be dissemination throughout the grant funding period.
- The project will be implemented in a complex inner-city diverse population.

Weaknesses

- This implementation project is based on years of development and commitment with talented people – how well will it generalize to other urban school systems.

1. Significance:

Strengths

- Children receive more mental health services in schools than through any other system. The prevalence of school children with mental health concerns, including significant suicidality is skyrocketing.
- [redacted] is a strong setting as its schools serve minority, low-income populations and it is also committed to implementing evidence-based practices to improve outcomes.
- Investigators accurately identify siloed approaches to behavioral health care with lack of coordination between mental health services and educators as a major need for an intervention focusing on interorganizational teamwork.
- This is a natural extension of TeamSTEPPS to a novel setting but one comparable to mental health settings where TeamSTEPPS has an important supportive evidence base.

Weaknesses

- None noted

2. Investigators:

Strengths

- Dr. Wolk has worked within school mental health services in Philadelphia since 2012 and is clearly connected with school system leadership in their development of policy related to school based behavioral health.
- The proposal is the next logical step in Dr. Wolk's research trajectory where she found CBT training alone was inadequate due to difficulty obtaining organization support and collaboration as well as direct teacher collaboration. Moreover, her F32 identified promise but clear barriers to this collocated model where the need to engage school personnel directly is need to further implement TeamSTEPPS.
- UPENN has 10 years' experience collaborating with the school system in developing professional development content.
- The resubmission now includes a Ph.D. level statistician.

Weaknesses

- None noted

3. Innovation:

Strengths

- This is a highly innovative expansion of TeamSTEPPS developed for general medical settings, to a collocated mental health in schools setting.
- This uses interorganizational team science in an innovative but challenging context.
- This application in an inner city setting is also innovative.

Weaknesses

- The ability to implement this intervention is based on a long term collaboration between an academic medical center and a community school system- will it generalize broadly?

4. Approach:

Strengths

- Investigators are using CIFR as a strong theoretical model to guide their project and identify the inner setting targets to improve overall adoption.
- Outcomes focus appropriately on feasibility and acceptability but they do include behavioral and educational outcomes.
- Direct observation methods are a strength.
- In contrast with Aim 1, Aim 3 adequately includes teacher perspectives.

Weaknesses

- 15 interviews in Aim 1 seems low, particularly for such a large school system. Also, it seems like there should be more direct interview of teachers- there are not enough teachers sampled and why aren't student/family members interviewed?

5. Environment:

Strengths

- This is an excellent environment. There is a strong academic medical center team working with an inner city school system which faces many challenges and issues with lack of resources.

Weaknesses

- None noted.

Protections for Human Subjects

Strengths

- Acceptable/no concerns. Investigators address concerns raised about individually identifiable information in Medicaid claims data and fears of damaging credibility of school members. Most of this argument was based on the experience of the investigators.

Weaknesses

- None noted.

Degree of Responsiveness:

Strengths

- TeamSTEPPS in school settings addresses primarily minority children with mental health needs in school settings and thereby addresses three AHRQ priority populations.
- Addressing AHRQ priorities of wrap around care that improves access to mental health care and support recovery.

Weaknesses

- None noted.

Inclusion of Women and Minority Subjects:

Strengths

- This is a considerable strength of the proposed project. There will be good gender representation in the qualitative portions of this study.

Weaknesses

- No concerns

Inclusion of AHRQ Priority Populations:

- Acceptable: This addresses under-resourced urban minority populations.

Budget and Period of Support:

Strengths

- Well developed and justified budget. No concerns.

Weaknesses

- No concerns

Authentication of Key Resources Used:

Resubmission Applications:

Strengths

- This resubmission presents comprehensive methods for integrating qualitative and quantitative results.
- The investigators addressed feasibility of advisory board meetings as much as possible.
- The advisory board includes parents of children receiving metal health services.
- They have confirmation that TeamSTEPPS training will occur during regular professional development time.

Weaknesses

- The resubmission did not address all issues raised, though many are inherent in addressing a study of this complexity.

THE FOLLOWING SECTIONS WERE PREPARED BY THE SCIENTIFIC REVIEW OFFICER TO SUMMARIZE THE OUTCOME OF DISCUSSIONS OF THE REVIEW COMMITTEE, OR REVIEWERS' WRITTEN CRITIQUES, ON THE FOLLOWING ISSUES:

RESUBMISSION NOTE: The application seems to be responsive to comments from the previous review.

PROTECTION OF HUMAN SUBJECTS: ACCEPTABLE

INCLUSION OF WOMEN PLAN: ACCEPTABLE

INCLUSION OF MINORITIES PLAN: ACCEPTABLE

INCLUSION OF CHILDREN PLAN: ACCEPTABLE

INCLUSION OF AHRQ PRIORITY POPULATIONS PLAN: ACCEPTABLE

COMMITTEE BUDGET RECOMMENDATIONS: The budget was recommended as requested

---

Footnotes for 1 R18 HS026862-01A1; PI Name: Wolk, Courtney Lynn Benjamin

+ Derived from the range of percentile values calculated for the study section that reviewed this application.
